# Home Cardiorespiratory Monitoring in Infants at Risk for Sudden Infant Death Syndrome (SIDS), Apparent Life-Threatening Event (ALTE) or Brief Resolved Unexplained Event (BRUE)

**DOI:** 10.3390/life12060883

**Published:** 2022-06-13

**Authors:** Chiara Sodini, Letizia Paglialonga, Giulia Antoniol, Serafina Perrone, Nicola Principi, Susanna Esposito

**Affiliations:** 1 Pediatric Clinic, Department of Medicine and Surgery, University of Parma, 43126 Parma, Italy; chiara.sodini@gmail.com (C.S.); letizia.paglialonga@gmail.com (L.P.); giulia.antoniol@gmail.com (G.A.); 2 Neonatology Unit, Department of Medicine and Surgery, University of Parma, 43126 Parma, Italy; serafina.perrone@unipr.it; 3 Università Degli Studi di Milano, 20122 Milan, Italy; nicola.principi@unimi.it

**Keywords:** ALTE, BRUE, cardiorespiratory monitoring, SIDS, sudden infant death syndrome, SUDS

## Abstract

Sudden infant death syndrome (SIDS) is defined as the sudden death of an infant younger than one year of age which remains unexplained after a thorough case investigation, including performance of a complete autopsy, examination of the death scene, and review of the clinical history. About 90% of SIDS occur before six months of age, the peak incidence is between two and four months, and the median age for death is elven weeks. The clinical, social, and economic relevance of SIDS, together with the evidence that prevention of this syndrome was possible, has significantly stimulated research into risk factors for the development of SIDS in the hope of being able to introduce new effective preventive measures. This narrative review discusses the potential relationships between apparent life-threatening events (ALTE) or brief resolved unexplained events (BRUE) and SIDS development, and when a home cardiorespiratory monitor is useful for prevention of these conditions. A literature analysis showed that home cardiorespiratory monitoring has been considered a potential method to identify not only ALTE and BRUE but SIDS also. ALTE and BRUE are generally due to underlying conditions that are not detectable in SIDS infants. A true relationship between these conditions has never been demonstrated. Use of home cardiorespiratory monitor is not recommended for SIDS, whereas it could be suggested for children with previous ALTE or severe BRUE or who are at risk of the development of these conditions. However, use of home cardiorespiratory monitors assumes that family members know the advantages and limitations of these devices after adequate education and instruction in their use.

## 1. Introduction

According to the National Institute for Child Health and Development of the USA, sudden infant death syndrome (SIDS) is defined as the sudden death of an infant younger than one year of age which remains unexplained after a thorough case investigation, including performance of a complete autopsy, examination of the death scene, and review of the clinical history [1]. SIDS is the major component of the group of the sudden unexpected infant deaths (SUIDs) that includes, together with SIDS, the infant deaths due to accidental suffocation in the sleeping environment and all of the other infant deaths of unknown causes. This last subgroup includes all of the unexpected infant deaths that are dependent on a defined underlying disease and that can mimic SIDS [2]. About 90% of SIDS occur before the six months of age, the peak incidence is between two and four months, and the median age for death is 11 weeks [3]. Moreover, it is more common among premature infants [4], low birth weight infants [5], siblings of SIDS victims [6], males [7] and in selected populations such as, in the USA, black and American/Alaskan native children [8,9].

SIDS is a dramatic condition that was not uncommon in the last century but that has significantly declined in recent years due to the introduction of preventive measures such as the use of safe sleeping conditions for infants [10,11,12]. In the USA, in 1990, when the American Academy of Pediatrics had not yet issued a recommendation for safe sleeping, SIDS rates were 130.3 per 100,000 live births. In 2019, about 27 years after the first recommendations [13,14], only 33.3 SIDS cases per 100,000 live births were reported. Despite this, SIDS remains the leading cause of death in children between one month and one year of age. Moreover, the risk of death from SIDS in the first 12 months of life is 20-fold higher than the risk of death due to any other cause during any of the following 17 years of life [15].

The clinical, social, and economic relevance of SIDS, together with the evidence that prevention of this syndrome was possible, has significantly stimulated research into risk factors for the development of SIDS in the hope of being able to introduce new effective preventive measures. In this regard, the supposition that significant acute and unexpected respiratory and cardiological alterations could lead to SIDS has strongly supported the use of home cardiorespiratory monitoring, at least in those children with a supposed or demonstrated risk of apparent life-threatening event (ALTE) or brief resolved unexplained event (BRUE). This narrative review discusses the potential relationships between ALTE and BRUE and SIDS development and the use of home cardiorespiratory monitors for prevention of these conditions. A formal systematic review or meta-analysis was not performed for the absence of a randomized controlled trial on these topics.

## 2. Present Hypothesis on Sudden Infant Death Syndrome (SIDS) Development

Current evidence seems to indicate that SIDS is due to the combination of several factors that act on vulnerable children during their first developmental periods. It is supposed that in utero environmental conditions and/or genetic abnormalities can cause brain alterations in the mechanisms that regulate respiratory and cardiac functions during the first months of life, limiting the development of protective mechanisms when a life-threatening event occurs. Polymorphisms of genes involved in the development and function of the autonomic nervous system have been found in up to 15% of SIDS cases [16]. Studies have shown that, compared to controls, SIDS children have more frequent significant abnormalities of both the serotonin transporter gene [17,18] and the monoamine oxidase gene [19,20], leading to decreased serotonergic receptor binding and reduced serotonin and tryptophan hydroxylase 2 levels in the brainstem with poor function of the autonomic nervous system. Moreover, alterations in genes encoding for cardiac ion channels [21,22], proteins involved in myocardial conduction [23], skeletal muscle sodium channels [24], several immune components (complement component C4, interleukin-10 promoter) [25,26], and heat shock proteins [27] have been reported. All of these findings suggest that SIDS prone children have significant differences in several body functions with poor response in front of life-threatening conditions. Among in utero environmental conditions, drug and alcohol abuse may play a role [28,29]. However, the most important in utero risk factor is the exposure to cigarette smoke or nicotine. Studies have shown that animals exposed to cigarette smoke or nicotine have significant alterations in the functions of the serotonin system and that this can lead to relevant alterations in the neuroregulation and cardiorespiratory activities [30]. In humans, it has been reported that exposure to tobacco smoke during fetal life attenuates recovery from hypoxia in preterm infants and significantly modifies heart function in both preterm and term infants [31,32,33]. On the other hand, about 70% of SIDS victims have deviations in 5-hydroxytryptamine binding in the medulla [34]. Moreover, prematurity and low birth weight infants, both conditions associated with SIDS [4,5], are strictly related to active or passive smoke exposure during pregnancy [35]. When extrinsic factors requiring protective physiologic mechanisms occur in children with these brain alterations, SIDS can develop. A prone sleep position is the most important extrinsic factor associated with SIDS. The evidence that in several countries the recommendation to avoid this sleeping position in the first year of life has been accompanied by a significant reduction in SIDS rate is per se a strong demonstration that the prone position has a role in SIDS development [10,11,12,13,14]. Several well conducted epidemiological studies have clearly shown that, compared to the supine position, the prone position is associated with a 1.7–12.9-fold increased risk of SIDS [36]. it was initially thought that any non-prone sleeping position could be protective, but later studies [37,38] have shown that despite it being significantly safer than the prone position, the side sleeping position was slightly less protective than the supine position, probably due to the possibility that children placed on their sides can roll to the prone position. Even the use of infant sleep positioners which are supposed to hold the baby on the side cannot be considered safe. One study reported the deaths of 13 infants kept on their side by means of a sleep positioner and that were then found prone or with the positioner against the face [39]. Several mechanisms to explain prone sleeping-related SIDS have been suggested [40,41]. Airways may be occluded by the flattening of the nose with distortion of the nasal cartilages or backward displacement of the tongue. Moreover, particularly when soft pillows and other soft bedding items are used, the rebreathing of carbon dioxide, cerebral hypoxia caused by constriction of arteries and overheating can play a role. The presence of a full stomach leading to an elevated diaphragm with reduced lung expansion may further favor SIDS. On the other hand, studies have shown that the supine position improves cerebral oxygenation [42]. Finally, the combination of prone sleep position and infection appears to significantly increase the risk of SIDS [43,44]. The common pathological findings of acute inflammatory change in the airways and lungs and other findings suggest alternative mechanisms underlying SIDS [45].

Table 1 summarizes the main risk factors associated with SIDS development.

## 3. Apparent Life-Threating Event (ALTE) and Brief Resolved Unexplained Event (BRUE): Potential Association with Sudden Infant Death Syndrome (SIDS)

In the first months of life, some children experience sudden episodes that can be, even if rarely, followed by death. They are named ALTE and BRUE, are generally unexpected, and because most of the cases are characterized by apnea, they are suspected of being a precursor to SIDS.

An ALTE is defined as a sudden event in an infant under the age of one year that is frightening to the observer who fears that the infant is dead and is characterized by the combination of apnea (central or occasionally obstructive), color change (usually cyanotic or pallid, but occasionally erythematous or plethoric), with a marked change in muscle tone (usually marked limpness), choking or gagging [46]. ALTE can occur during sleep, when the infant is awake, or during feeding. Cardiorespiratory support may be necessary, ranging from basic to full advanced life support. Although ALTE represent a relatively common event in emergency departments, reaching 0.6–0.8% of the total admissions of children below 12 months of age [47], the precise incidence of this condition is unknown. Hospital records indicate a frequency varying from 0.46 to 10.0 per 1000 live births, depending on the methods used to identify the episodes. When parents’ responses to questions regarding already solved episodes were used, the highest frequency values were recorded [48,49,50,51]. It is, however, unquestionable that the SIDS prevention campaigns directed at new parents together with a heightened awareness among pediatricians are probably contributing to the increased detection of ALTE [52].

ALTE is associated with or caused by a variety of diseases that can be identified in more than 50% of the cases after a careful history, physical examination, and appropriate laboratory evaluation. Gastroesophageal reflux (GER)/laryngospasm, neurologic problems such as seizures, and respiratory infections are the most common underlying diseases. Child abuse is less common (0.4% to 11%). Infrequent causes are cardiac diseases, upper airway obstruction, metabolic disorders, anaphylaxis, and bacterial infections (including urinary tract infection), each accounting for 0.5% to 3% of ALTE. Even rarer causes are disorders of ventilatory control, toxic ingestions, and other miscellaneous conditions (Table 2). However, more than 30% of the cases remain undetermined, and the event is then defined as idiopathic ALTE [53,54,55,56,57]. As some of these children may die, it is possible that a portion could be classified as SIDS after appropriate investigations.

In 2016 the AAP proposed the replacing of the term ALTE with the new acronym BRUE [58]. The intent was both to leave the characterization of the episode to the clinician and to reduce the caregiver’s perception of severity, with the aim of improving clinical care and management. Specifically, clinicians should use the term BRUE to describe an event occurring in an infant < 1 year of age when the observer reports a sudden, brief, and now resolved episode of one or more of the following: cyanosis or pallor; absent, decreased, or irregular breathing; marked change in tone (hyper or hypotonia); and altered level of responsiveness. Moreover, clinicians should diagnose a BRUE only when the infant is asymptomatic on presentation and when there is no explanation for the episode after a focused history and physical examination have been conducted. In any case, BRUE might hide an undiagnosed serious condition or an increased probability of recurrence; therefore, an accurate stratification from low to high risk is also recommended by the AAP guidelines. To be designated lower risk, the following criteria should be met: age > 60 days; prematurity (gestational age 32 or more weeks and postconceptional age 45 or more weeks); first BRUE (no previous BRUE ever and not occurring in clusters); duration of event < 1 min (and typically < 20–30 s); no CPR required by trained medical provider (resuscitation by an untrained caregiver is not used as an independent marker for medically significant BRUE); no concerning historical feature like social risk factors for child abuse, respiratory illness or exposure, recent injury, other symptoms in days preceding the event (fever, fussiness, diarrhea or decreased intake), administration or access to medications, history of episodic vomiting or lethargy, developmental delay or congenital anomalies, and family history of BRUE or sudden unexplained death in a sibling; no concerning physical examination findings that include any signs of injury, altered sensorium, fever or toxic appearance, respiratory distress, heart murmur or gallop, and decreased pulse [58]. Infants who meet all these criteria for low-risk BRUE, who are asymptomatic and have no concerning features identified on history and physical examination, require minimal additional evaluation or observation. Infants who do not meet these low-risk criteria are thought to be at higher risk for a serious underlying disorder, recurrent event or adverse outcome and require more extensive evaluation. If there are clinical features that suggest a specific diagnosis (e.g., upper respiratory tract infection or child abuse), the evaluation may be targeted toward that concern.

In 2017, the Italian Society of Pediatrics updated its own guidelines and accepted the new acronym BRUE for lower-risk idiopathic episodes, while keeping the term ALTE for severe idiopathic episodes that are only solved after resuscitation maneuvers and related to patient instability, prematurity (<32 weeks of gestational age and <43 weeks of post-conceptional age), age below two months, need for resuscitation maneuvers, recurrence, and poor family compliance [51,53]. Clinical evaluation of children with BRUE showed that, in the great majority, children with this diagnosis have very mild disease that does not require hospital admission for further tests [59,60].

Episodes of ALTE are reported in the clinical history of some children with SIDS [61]. This explains why for many years it was thought that the two conditions could be related. Despite this, several findings seem to suggest that ALTE and severe BRUE are not precursors of SIDS [1,53,62,63,64]. First of all, the evidence is that only a low number of children experiencing ALTE or BRUE die from SIDS (0–7%). Moreover, while the incidence of SIDS has decreased after the introduction of recommendations for safe infant sleeping, the incidence of ALTE has remained unchanged or even increased [10,11,12,13,14]. ALTE, BRUE and SIDS, although disorders of the first year of life, differ significantly in age-at-event. The mean age of SIDS is 10 weeks later than that of ALTE. Unlike SIDS, which occurs during sleeping, ALTE episodes occur during wakefulness. Apnea and bradycardia that characterize ALTE are less frequent in the early morning, when SIDS is more common. The incidence of ALTE is equally distributed between boys and girls, whereas SIDS occurs more frequently in boys. The age of the mother whose children had experienced ALTE is generally older than that of a mother whose infants had succumbed to SIDS [1,62,63,64].

Finally, despite some risk factors for ALTE, BRUE and SIDS being common, the prevalence of each of them seems to be different. A study that has analyzed the rate of the most common ALTE risk variables (male predominance, gestational age, low birth weight, very low birth weight, incidence of small for gestational age, age at the event, multiparity, maternal age, and smoking) reported that four of them: low birth weight and SGA at birth, teenage pregnancies, and a younger infant age, were significantly more common in children with ALTE than in those with SIDS [65]. Moreover, when frequency of some risk factors for SIDS development were evaluated in children with ALTE or SIDS, it was found that the prevalence of prone sleeping, smoking, bottle-feeding, low maternal age and education were higher amongst SIDS compared with ALTE cases. Interestingly, the prevalence of these variables was quite similar in ALTE cases and in matched healthy controls [66].

However, it cannot be excluded that a small number of children with a previous episode of idiopathic ALTE or idiopathic and very severe BRUE can later develop SIDS. Genetic studies involving serotonin transporter and monoamino oxidase A in children with secondary ALTE and idiopathic ALTE have shown that differences exist between the two conditions and that those identified in idiopathic ALTE resemble those in SIDS [65]. This has suggested that idiopathic ALTE may be a precursor of SIDS, particularly when other factors able to induce the relevant modification of cardiorespiratory activity can simultaneously occur. In this regard, a prone sleeping position and smoking during pregnancy may play a role. Edner et al. have shown that 33.3% of infants with ALTE who subsequently died of SIDS had the dual risk factors of prone sleep position and late prenatal smoke exposure, compared with 13.3% of ALTE survivors [66]. The importance of exposure to cigarette smoke during pregnancy seems confirmed by a study in which respiratory characteristics and arousal of children who had ALTE were compared with those of healthy children and SIDS patients [61]. It was found that, despite children with ALTE and nonsmoking mothers having fewer total arousals, cortical arousals, and subcortical activations than normal controls, patterns of spontaneous arousals differed significantly from those of future SIDS victims. On the contrary, in children with ALTE and smoking mothers, arousal and respiratory characteristics were quite similar to those of future SIDS victims, suggesting some common abnormalities in brainstem function that could be implicated in SIDS development. On the other hand, it seems likely that women smoking during pregnancy continue to smoke after delivery. As smoke exposure in the first months of life may exacerbate viral and bacterial infections and can have effects on the immune system and the CNS, subsequent SIDS in genetically predisposed infants can be further favored.

## 4. Use of Home Cardiorespiratory Monitoring in Sudden Infant Death Syndrome (SIDS) Prevention

Initially, it was thought that because SIDS was more common in children at risk of apnea and bradycardia, accurate monitoring of cardiorespiratory functions could be a valid measure to prevent SIDS. Presently, the use of cardiorespiratory monitors for SIDS prevention is not recommended, as several studies have clearly demonstrated that these devices are not an effective tool in this regard, regardless of the presence of risk factors for SIDS development [67,68]. Conventional monitors detect central apnea but not obstructive apnea in the absence of bradycardia or a relevant drop in oxygen saturation that, on the contrary, seems to be clinically relevant in SIDS development [69]. The relationship between apnea and SIDS has been denied since 1986. In a group of 180,017 infants, the incidence of SIDS was three times higher than that of proven infant apnea. Only five babies with previous proven apnea died from SIDS. Statistical analysis reveals that these five events cannot be explained as random coincidences as they represented 3% of 163 proved apnea cases, but only 1% of 503 SIDS cases [70]. Further confirmation of the lack of relationships between apnea and bradycardia commonly found in children with ALTE and SIDS was obtained with the Collaborative Home Infant Monitoring Evaluation (CHIME) study that showed that cardiorespiratory events were quite common, with similar frequency and severity in previously heathy infants and in those considered at risk of SIDS. Increased risk of at least one extreme event was demonstrated in only preterm infants and only until about 43 weeks’ post conceptional age, suggesting that they were not likely to be precursors to SIDS [71].

However, home cardiorespiratory monitor use can be suggested in a selected group of children with previous ALTE or severe BRUE or ones that are at risk for these conditions (i.e., for non-SIDS underlying pathology). In these cases, the rapid identification of an acute respiratory or cardiac problem can be important to identify underlying severe conditions that can cause ALTE and lead to permanent alterations or even death. Over the years, several studies have been conducted to assess the usefulness of event recording in identifying the mechanisms underlying ALTE, detecting sporadic episodes which could be missed during a brief hospitalization. In a 1993 study carried out by Poets et al. [72], a group of 94 infants with a history of two or more ALTE events was monitored for approximately one month using a device that recorded breathing movements, ECG, heart rate, pulse oximeter saturation and the plethysmographic or pulse waveforms from the oximeter for approximately 20 min around the alarm triggered by a fall in transcutaneous partial pressure of oxygen. Analysis of the recordings identified potentially preventable mechanisms of ALTE in about 63% of the infants. Specifically, the following underlying mechanisms were identified: changes in skin perfusion in the absence of hypoxia, hypoxia caused by an epileptic seizure, hypoxia induced by suffocation by a parent, and Munchausen by proxy. Later, Marcus and Hamer [73] showed an association between gastroesophageal reflux and isolated bradycardias, not preceded by apnea, especially when asystole was documented. However, before recommending home monitor use it should be remembered that some problems can arise [73].

Home monitoring can have significant psychologic benefits, particularly for families with a previous experience of SIDS, because it can decrease feelings of guilt and anxiety. In some cases, however, parents may feel falsely reassured and reduce personal control of children with problems [47,70]. False alarms can occur, and this can cause anxiety and psychological stress in parents. Finally, in infants at risk whose monitoring is recommended, despite the comfort that the continuous monitoring itself can give, parents can experience emotional stress, frustration and depression because of the feeling that their infant is less healthy than others. In every case, home monitoring can create an overdependence, and its discontinuation can be hard to accept by the family, who could insist that they continue the use of the monitor even when the indication is over [47]. This could explain why the recommendations from the American Academy of Pediatrics for use of home cardiorespiratory monitors use does not include infants with previous ALTE or severe BRUE, but limits monitoring to premature infants who are at high risk of recurrent episodes of apnea, bradycardia, and hypoxemia after hospital discharge until the 43 weeks postmenstrual age or after the cessation of extreme episodes, whichever comes last [74]. Moreover, home apnea monitoring may be warranted for infants who are technology dependent (tracheostomy, continuous positive airway pressure), have unstable airways, have rare medical conditions affecting regulation of breathing, or have symptomatic chronic lung disease. Italian guidelines published in 2017 are more extensive and recommend home monitoring prescription for infants with severe or recurrent ALTE and for preterm infants with a clinical history of ALTE, especially with a postconceptional age < 43 weeks, with a duration of at least six weeks [52] (Table 3).

However, when home monitoring is performed regardless of the indication (i.e., SIDS, ALTE or BRUE), a reliable and safe monitoring device should be preferred. Currently, the machines are smaller and more manageable than those used several years ago; they usually allow detection of vital health signs via body-worn wireless sensor integrated wearable; a wirelessly connected base station collects health-related information and sends it back to both the parents’ cell phone and the internet cloud; transmitted data are stored in the cloud, which is accessible from anywhere; and, finally, parents get a notification directly from the base station as well as from cell phones in any unexpected decline in the infant’s vitals beyond the thresholds [75]. Despite significant improvement in sensitivity and specificity, false alarms are not excluded. The last generation of infant monitors are not recommended, however; i.e., those based on smartphone applications integrated with sensors built into socks, onesies, buttons, leg bands, and diaper clips. They display infants’ respiration, pulse rate, and/or blood oxygen saturation and generate alarms for apnea, tachycardia, bradycardia, and/or desaturation. They are not considered and sold as medical devices, and are not currently subject to regulation by the US Food and Drug Administration or by the European Agency of Medicine [76]. Their safety, accuracy and effectiveness have not yet been evaluated by appropriate scientific studies, and initial evaluations indicate that they have very poor sensitivity for both hypoxemia and bradycardia [77,78].

## 5. Conclusions

SIDS has been largely studied and some of the factors that are associated with its development have been identified. Efforts to eliminate these factors have been successful, and there is no doubt that many cases of SIDS have been prevented. However, biologic mechanisms that lead to SIDS are not precisely defined, and this does not allow for the accurate identification of children at risk and the implementation of measures capable of avoiding all SIDS cases. Conditions such as ALTE and BRUE that in the most severe cases are associated with apnea, bradycardia and other clinical manifestations that can lead to death have been considered as potential SIDS precursors. Cardiorespiratory monitoring has been considered a potential method to identify not only ALTE and BRUE, but SIDS as well. Unfortunately, ALTE and BRUE are generally due to underlying conditions that are not detectable in SIDS infants. A true relationship between these conditions has never been demonstrated. Only a minority of ALTE are idiopathic, and for these cases a possible relationship with SIDS development could be supposed. The use of a home cardiorespiratory monitor is not recommended for SIDS. We think that as reported in the Parma consensus, home cardiorespiratory monitors could be suggested for children with previous ALTE or severe BRUE or who are at risk of developing these conditions. The aim of this use is the early identification of apnea, bradycardia or hypoxemia in subjects at increased risk of significant clinical problems in cases of persistent cardiorespiratory deficits. However, the use of home cardiorespiratory monitors assumes that family members know the advantages and limitations of these devices after adequate education and information. Further studies aimed to investigate the risk factors for SIDS and the pathophysiological mechanisms underlying these extreme events would be helpful to more clearly define the possible applications of home monitoring in the management of patients at increased risk of SIDS.

## Figures and Tables

**Table 1:** Main risk factors associated with SIDS development.

Gender Factors
Ethnic differences (in the United States, risks are highest in Black and American Ind- ian/Alaskan native infants, followed by non- Hispanic white infants, and are lowest in Asian/Pacific islander and Hispanic infants) Gender—male slightly greater than female Climate-higher risk in cold versus warm months
**Maternal and Antenatal Factors**
Smoking, illicit drugs Young, single parent, no high school degree Late or no prenatal care Poor gestational weight gain Anemia Pregnancy complications (placenta previa, abruption, premature rupture of membrane) Infection
**Neonatal Factors**
Prematurity Low birth weight Small for gestational age
**Post-Neonatal Factors**
Bed-sharing Prone sleep position Sleep with bedding accessories such as loose blankets and pillows Overheating Infection

**Table 2:** Main etiology of apparent life-threating event (ALTE).

**Idiopathic (approximately 50%** **Gastrointestinal (most common, up to 50% of diagnosed cases)** Gastroesophageal reflux Gastric volvulus Intussusception Swallowing abnormalities Other GI abnormalities **Neurologic (approximately 30%)** Seizure disorder Febrile seizure CNS bleeding Neurologic conditions affecting respiration (Budd-Chiari syndrome, hindbrain malformation, brainstem malformation) Vasovagal reflexes Hydrocephalus CNS infection Ventriculoperitoneal shunt malfunction **Respiratory (approximately 20%)** Respiratory compromise from infection, respiratory syncytial virus, pertussis, mycoplasma, croup, other pneumonias Obstructive sleep apnoea Breath-holding spells Conditions affecting respiratory control (prematurity, central hypoventilation) Vocal cord abnormalities, adenoid vegetations Laryngotracheomalacia Airway obstruction resulting from congenital abnormalities Foreign-body aspiration	**Cardiac (up to 5%)** Arrhythmia Long QT syndrome Wolff-Parkinson-White syndrome Congenital heart disease Myocarditis Cardiomyopathy **Metabolic abnormalities (less than 5%)** Inborn errors of metabolism Endocrine, electrolyte disorders Other infections Urinary tract infection Sepsis **Child abuse (less than 5%)** Munchausen syndrome by proxy (suffocation, intentional salt poisoning, medication overdose, physical abuse, head injury) Smothering (unintentional or intentional) **Other** Food allergy (uncommon) Anaphylaxis Medication (prescription, over the counter, herbal remedies)

**Table 3:** Home monitoring recommendations from different scientific societies.

Recommendation	American Academy of Pediatrics [74]	Italian Guidelines [54]	Parma Consensus [52]
**Home monitoring recommendation**	Not recommended for prevention of SIDS. Suggested only for selected preterm children at risk (see text)	- Sever or recurrent ALTE - ALTE in preterm infants	- Recurrent ALTE - ALTE in preterm and postconceptional age < 43 weeks
**Duration**	Until the 43 weeks postmenstrual age or after the cessation of extreme episodes, whichever comes last	- At least six weeks - Until the 43 weeks of postconceptional age	- Until the 43rd week of postconceptional age

## Data Availability

All the data are included in the manuscript.

## References

[1] Willinger M., James L.S., Catz C. (1991). Defining the sudden infant death syndrome (SIDS): Deliberations of an expert panel convened by the National Institute of Child Health and Human Development. Pediatr. Pathol..

[2] Centers for Disease Control and Prevention (CDC) Sudden Unexpected Infant Death and Sudden Infant Death Syndrome. https://www.cdc.gov/sids/data.htm.

[3] Hoffman H.J., Damus K., Hillman L., Krongrad E. (1988). Risk factors for SIDS. Results of the National Institute of Child Health and Human Development SIDS Cooperative Epidemiological Study. Ann. N. Y. Acad. Sci..

[4] Malloy M.H., Hoffman H.J. (1995). Prematurity, sudden infant death syndrome, and age of death. Pediatrics.

[5] Bigger H.R., Silvestri J.M., Shott S., Weese-Mayer D.E. (1998). Influence of increased survival in very low birth weight, low birth weight, and normal birth weight infants on the incidence of sudden infant death syndrome in the United States: 1985–1991. J. Pediatr..

[6] Carpenter R.G., Waite A., Coombs R.C., Daman-Willems C., McKenzie A., Huber J., Emery J.L. (2005). Repeat sudden unexpected and unexplained infant deaths: Natural or unnatural?. Lancet.

[7] Carpenter R.G., Irgens L.M., Blair P.S., England P.D., Fleming P., Huber J., Jorch G., Schreuder P. (2004). Sudden unexplained infant death in 20 regions in Europe: Case control study. Lancet.

[8] Mathews T.J., Menacker F., MacDorman M.F. (2004). Centers for Disease Control and Prevention, National Center for Health Statistics. Infant mortality statistics from the 2002 period: Linked birth/infant death data set. Natl. Vital. Stat. Rep..

[9] Moon R.Y. (2016). Task Force on Sudden Infant Death Syndrome. SIDS and Other Sleep-Related Infant Deaths: Evidence Base for 2016 Updated Recommendations for a Safe Infant Sleeping Environment. Pediatrics.

[10] Markestad T., Skadberg B., Hordvik E., Morild I., Irgens L.M. (1995). Sleeping position and sudden infant death syndrome (SIDS): Effect of an intervention programme to avoid prone sleeping. Acta Paediatr..

[11] Willinger M., Hoffman H.J., Hartford R.B. (1994). Infant sleep position and risk for sudden infant death syndrome: Report of meeting held January 13 and 14 1994, National Institutes of Health, Bethesda MD. Pediatrics.

[12] Kiechl-Kohlendorfer U., Peglow U.P., Kiechl S., Oberaigner W., Sperl W. (2001). Epidemiology of sudden infant death syndrome (SIDS) in the Tyrol before and after an intervention campaign. Wien Klin. Wochenschr..

[13] American Academy of Pediatrics (1992). AAP Task Force on Infant Positioning and SIDS. Pediatrics.

[14] American Academy of Pediatrics (1996). AAP Task Force on Infant Positioning and SIDS. Positioning and sudden infant death syndrome (SIDS): Update. Pediatrics.

[15] Roehler D.R., Batra E.K., Quinlan K.P. (2019). Comparing the Risk of Sudden Unexpected Infant Death to Common Causes of Childhood Injury Death. J. Pediatr..

[16] Weese-Mayer D.E., Berry-Kravis E.M., Zhou L. (2004). Sudden infant death syndrome: Case-control frequency differences at genes pertinent to early autonomic nervous system embryologic development. Pediatr. Res..

[17] Narita N., Narita M., Takashima S., Nakayama M., Nagai T., Okado N. (2001). Serotonin transporter gene variation is a risk factor for sudden infant death syndrome in the Japanese population. Pediatrics.

[18] Opdal S.H., Vege A., Rognum T.O. (2008). Serotonin transporter gene variation in sudden infant death syndrome. Acta Paediatr..

[19] Klintschar M., Heimbold C. (2012). Association between a functional polymorphism in the MAOA gene and sudden infant death syndrome. Pediatrics.

[20] Courts C., Grabmüller M., Madea B. (2013). Monoamine oxidase A gene polymorphism and the pathogenesis of sudden infant death syndrome. J. Pediatr..

[21] Johannsen E.B., Baughn L.B., Sharma N., Zjacic N., Pirooznia M., Elhaik E. (2021). The Genetics of Sudden Infant Death Syndrome-Towards a Gene Reference Resource. Genes.

[22] Keywan C., Poduri A.H., Goldstein R.D., Holm I.A. (2021). Genetic Factors Underlying Sudden Infant Death Syndrome. Appl. Clin. Genet..

[23] Van Norstrand D.W., Asimaki A., Rubinos C., Dolmatova E., Srinivas M., Tester D.J., Saffitz J.E., Duffy H.S., Ackerman M.J. (2012). Connexin43 mutation causes heterogeneous gap junction loss and sudden infant death. Circulation.

[24] Männikkö R., Wong L., Tester D.J., Thor M.G., Sud R., Kullmann D.M., Sweeney M.G., Leu C., Sisodiya S.M., FitzPatrick D.R. (2018). Dysfunction of NaV1.4, a skeletal muscle voltage-gated sodium channel, in sudden infant death syndrome: A case-control study. Lancet.

[25] Opdal S.H., Vege A., Stave A.K., Rognum T.O. (1999). The complement component C4 in sudden infant death. Eur. J. Pediatr..

[26] Opdal S.H., Opstad A., Vege A., Rognum T.O. (2003). IL-10 gene polymorphisms are associated with infectious cause of sudden infant death. Hum. Immunol..

[27] Courts C., Grabmüller M., Madea B. (2013). Functional single-nucleotide variant of HSPD1 in sudden infant death syndrome. Pediatr. Res..

[28] Ward S.L., Bautista D., Chan L., Derry M., Lisbin A., Durfee M.J., Mills K.S., Keens T.G. (1990). Sudden infant death syndrome in infants of substance-abusing mothers. J. Pediatr..

[29] Strandberg-Larsen K., Grønboek M., Andersen A.M., Andersen P.K., Olsen J. (2009). Alcohol drinking pattern during pregnancy and risk of infant mortality. Epidemiology.

[30] Li A., Darnall R.A., Dymecki S., Leiter J.C., Duncan J.R., Byard R.W. (2018). Animal Models: Illuminating the Pathogenesis of Sudden Infant Death Syndrome. SIDS Sudden Infant and Early Childhood Death: The Past, the Present and the Future.

[31] Schneider J., Mitchell I., Singhal N., Kirk V., Hasan S.U. (2008). Prenatal cigarette smoke exposure attenuates recovery from hypoxemic challenge in preterm infants. Am. J. Respir. Crit. Care Med..

[32] Thiriez G., Bouhaddi M., Mourot L., Nobili F., Fortrat J.O., Menget A., Franco P., Regnard J. (2009). Heart rate variability in preterm infants and maternal smoking during pregnancy. Clin. Auton. Res..

[33] Fifer W.P., Fingers S.T., Youngman M., Gomez-Gribben E., Myers M.M. (2009). Effects of alcohol and smoking during pregnancy on infant autonomic control. Dev. Psychobiol..

[34] Haynes R.L., Duncan J.R., Byard R.W. (2018). Biomarkers of Sudden Infant Death Syndrome (SIDS) Risk and SIDS Death. SIDS Sudden Infant and Early Childhood Death: The Past, the Present and the Future.

[35] Salihu H.M., Wilson R.E. (2007). Epidemiology of prenatal smoking and perinatal outcomes. Early Hum. Dev..

[36] American Academy of Pediatrics (2000). Task Force on Infant Sleep Position and Sudden Infant Death Syndrome Changing concepts of sudden infant death syndrome: Implications for infant sleeping environment and sleep position. Pediatrics.

[37] Mitchell E.A., Scragg R.K. (1994). Observations on ethnic differences in SIDS mortality in New Zealand. Early Hum. Dev..

[38] Fleming P.J., Blair P.S., Bacon C., Bensley D., Smith I., Taylor E., Berry J., Golding J., Tripp J. (1996). Environment of infants during sleep and risk of the sudden infant death syndrome: Results of 1993–5 case-control study for confidential inquiry into stillbirths and deaths in infancy. Br. Med. J..

[39] Centers for Disease Control and Prevention (CDC) (2012). Suffocation deaths associated with use of infant sleep positioners--United States, 1997–2011. MMWR Morb. Mortal. Wkly. Rep..

[40] Sperhake J., Jorch G., Bajanowski T. (2018). The prone sleeping position and SIDS. Historical aspects and possible pathomechanisms. Int. J. Legal. Med..

[41] Byard R.W., Bright F., Vink R. (2018). Why is a prone sleeping position dangerous for certain infants?. Forensic. Sci. Med. Pathol..

[42] Fyfe K.L., Yiallourou S.R., Wong F.Y., Odoi A., Walker A.M. (2014). Horne RS. Cerebral oxygenation in preterm infants. Pediatrics.

[43] Ponsonby A.L., Dwyer T., Gibbons L.E., Cochrane J.A., Wang Y.G. (1993). Factors potentiating the risk of sudden infant death syndrome associated with the prone position. N. Engl. J. Med..

[44] Helweg-Larsen K., Lundemose J.B., Oyen N., Skjaerven R., Alm B., Wennergren G., Markestad L.M., Irgens L.M. (1999). Interactions of infectious symptoms and modifiable risk factors in sudden infant death syndrome. The Nordic epidemiological SIDS study. Acta Paediatr..

[45] Goldwater P.N. (2020). SIDS, prone sleep position and infection: An overlooked epidemiological link in current SIDS research? Key evidence for the “Infection Hypothesis”. Med. Hypotheses.

[46] National Institutes of Health Infantile Apnea and Home Monitoring. Consensus Development Conference Statement September 29–October 1, 1986. https://consensus.nih.gov/1986/1986infantapneamonitoring058html.htm.

[47] Fu L.Y., Moon R.Y. (2007). Apparent Life-threatening Events (ALTEs) and the Role of Home Monitors. Pediatr. Rev..

[48] Wennergren G., Milerad J., Lagercrantz H., Karlberg P., Svenningsen N.W., Sedin G., Andersson D., Grögaardet J., Bjue J. (1987). The epidemiology of sudden infant death syndrome and attacks of lifelessness in Sweden. Acta Paediatr..

[49] Polberger S., Svenningsen N.W. (1985). Early neonatal sudden infant death and near death of fullterm infants in maternity wards. Acta Paediatr..

[50] Davis N., Bakke K. (1983). Evaluation of a home apnea monitoring program. Perinatol. Neonatol..

[51] Mandell F. (1981). Cot death among children of nurses. Observations of breathing patterns. Arch. Dis. Child..

[52] Prezioso G., Perrone S., Biasucci G., Pisi G., Fainardi V., Strisciuglio C., Marzano F.N., Moretti S., Pisani F., Tchana B. (2021). Management of Infants with Brief Resolved Unexplained Events (BRUE) and Apparent Life-Threatening Events (ALTE): A RAND/UCLA Appropriateness Approach. Life.

[53] Fu L.Y., Moon R.Y. (2012). Apparent life-threatening events: An update. Pediatr. Rev..

[54] Piumelli R., Davanzo R., Nassi N., Salvatore S., Arzilli C., Peruzzi M., Agosti M., Palmieri A., Paglietti M.G., Nosetti L. (2017). Apparent Life-Threatening Events (ALTE): Italian guidelines. Ital. J. Pediatr..

[55] Tieder J.S., Cowan C.A., Garrison M.M., Christakis D.A. (2008). Variation in inpatient resource utilization and management of apparent life-threatening events. J. Pediatr..

[56] Kiechl-Kohlendorfer U., Hof D., Peglow U.P., Traweger-Ravanelli B., Kiechl S. (2005). Epidemiology of apparent life threatening events. Arch. Dis. Child..

[57] McGovern M.C., Smith M.B. (2004). Causes of apparent life threatening events in infants: A systematic review. Arch. Dis. Child..

[58] Tieder J.S., Bonkowsky J.L., Etzel R.A., Franklin W.H., Gremse D.A., Herman B., Katz E.S., Krilov L.R., Merritt J.L., Norlin C. (2016). Brief Resolved Unexplained Events (Formerly Apparent Life-Threatening Events) and Evaluation of Lower-Risk Infants. Pediatrics.

[59] Bochner R., Tieder J.S., Sullivan E., Hall M., Stephans A., Mittal M.K., Singh N., Delanry A., Harper B., Shastri N. (2021). Explanatory Diagnoses Following Hospitalization for a Brief Resolved Unexplained Event. Pediatrics.

[60] Evers K.S., Wellmann S., Donner B.C., Ritz N. (2021). Apparent life-threatening events and brief resolved unexplained events: Management of children at a Swiss tertiary care center. Swiss. Med. Wkly..

[61] Franco P., Montemitro E., Scaillet S., Groswasser J., Kato I., Lin J.S., Villa M.P. (2011). Fewer spontaneous arousals in infants with apparent life-threatening event. Sleep.

[62] (1987). National Institutes of Health Consensus Development Conference on Infantile Apnea and Home Monitoring, Sept 29 to Oct 1, 1986. Pediatrics.

[63] Sahewalla R., Gupta D., Kamat D. (2016). Apparent Life-Threatening Events: An Overview. Clin. Pediatr..

[64] Gray C., Davies F., Molyneux E. (1999). Apparent life-threatening events presenting to a pediatric emergency department. Pediatr. Emerg. Care.

[65] Esani N., Hodgman J.E., Ehsani N., Hoppenbrouwers T. (2008). Apparent life-threatening events and sudden infant death syndrome: Comparison of risk factors. J. Pediatr..

[66] Edner A., Wennborg M., Alm B., Lagercrantz H. (2007). Why do ALTE infants not die in SIDS?. Acta Paediatr..

[67] Filonzi L., Magnani C., Nosetti L., Nespoli L., Borghi C., Vaghi M., Nonnis Marzano F. (2012). Serotonin transporter role in identifying similarities between SIDS and idiopathic ALTE. Pediatrics.

[68] Behnam-Terneus M., Clemente M. (2019). SIDS, BRUE, and Safe Sleep Guidelines. Pediatr. Rev..

[69] Akbarian S., Montazeri Ghahjaverestan N., Yadollahi A., Taati B. (2020). Distinguishing Obstructive Versus Central Apneas in Infrared Video of Sleep Using Deep Learning: Validation Study. J. Med. Internet Res..

[70] Davis N., Bossung-Sweeney L., Peterson D.R. (1986). Epidemiological comparisons of sudden infant death syndrome with infant apnoea. Aust. Paediatr. J..

[71] Ramanathan R., Corwin M.J., Hunt C.E., Lister G., Tinsley L.R., Baird T., Silvestri J.M., Crowell D.H., Hufford D., Martin R.J. (2001). Cardiorespiratory events recorded on home monitors: Comparison of healthy infants with those at increased risk for SIDS. JAMA.

[72] Poets C.F., Samuels M.P., Noyes J.P., Hewertson J., Hartmann H., Holder A., Southall D.P. (1993). Home event recordings of oxygenation, breathing movements, and heart rate and rhythm in infants with recurrent life-threatening events. J. Pediatr..

[73] Marcus C.L., Hamer A. (1999). Significance of isolated bradycardia detected by home monitoring. J. Pediatr..

[74] Dangerfield M.I., Ward K., Davidson L., Adamian M. (2017). Initial Experience and Usage Patterns with the Owlet Smart Sock Monitor in 47,495 Newborns. Glob. Pediatr. Health.

[75] American Academy of Pediatrics Committee on Fetus and Newborn (2003). Apnea, sudden infant death syndrome, and home monitoring. Pediatrics.

[76] Chen H., Xue M., Mei Z., Bambang Oetomo S., Chen W. (2016). A Review of Wearable Sensor Systems for Monitoring Body Movements of Neonates. Sensors.

[77] Bonafide C.P., Jamison D.T., Foglia E.E. (2017). The Emerging Market of Smartphone-Integrated Infant Physiologic Monitors. JAMA.

[78] Bonafide C.P., Localio A.R., Ferro D.F., Orenstein E.W., Jamison D.T., Lavanchy C., Foglia E.E. (2018). Accuracy of Pulse Oximetry-Based Home Baby Monitors. JAMA.

